# The Use of Optimal Treatment for DLBCL Is Improving in All Age Groups and Is a Key Factor in Overall Survival, but Non-Clinical Factors Influence Treatment

**DOI:** 10.3390/cancers11070928

**Published:** 2019-07-02

**Authors:** Nicole Wong Doo, Victoria M. White, Kara Martin, Julie K. Bassett, H. Miles Prince, Simon J. Harrison, Michael Jefford, Ingrid Winship, Jeremy L. Millar, Roger L. Milne, John F. Seymour, Graham G. Giles

**Affiliations:** 1 Cancer Epidemiology Division, Cancer Council Victoria, Melbourne, VIC 3004, Australia; 2 Concord Repatriation General Hospital, Sydney Medical School, University of Sydney, Sydney, NSW 2139, Australia; 3 Concord Clinical School, University of Sydney, Concord, NSW 2139, Australia; 4 School of Psychology, Faculty of Health, Deakin University, Geelong, VIC 3220, Australia; 5 Centre for Behavioural Research in Cancer, Cancer Council Victoria, Melbourne, VIC 3004, Australia; 6 Department of Haematology, Peter MacCallum Cancer Centre & Royal Melbourne Hospital, Melbourne, VIC 3000, Australia; 7 Sir Peter MacCallum Department of Oncology, University of Melbourne, Parkville, VIC 3010, Australia; 8 Department of Medical Oncology, Peter MacCallum Cancer Centre, Melbourne, VIC 3000, Australia; 9 Genomic Medicine and Family Cancer Clinic, Royal Melbourne Hospital, Parkville, VIC 3050, Australia; 10 Department of Medicine, The University of Melbourne, Parkville, VIC 3010, Australia; 11 Alfred Health Radiation Oncology, Alfred and LaTrobe Regional Hospital, Melbourne, VIC 3004, Australia; 12 School of Public Health and Preventive Medicine, Monash University, Melbourne, VIC 3004, Australia; 13 Centre for Epidemiology and Biostatistics, Melbourne School of Population and Global Health, The University of Melbourne, Parkville, VIC 3010, Australia; 14 Precision Medicine, School of Clinical Sciences at Monash Health, Monash University, Clayton, VIC 3800, Australia

**Keywords:** diffuse large B cell lymphoma, chemotherapy, epidemiologic studies, patterns of care, cancer survival

## Abstract

*Introduction*: Diffuse large B cell lymphoma (DLBCL) is an aggressive form of non-Hodgkin lymphoma for which a cure is usually the therapeutic goal of optimal treatment. Using a large population-based cohort we sought to examine the factors associated with optimal DLBCL treatment and survival. *Methods*: DLBCL cases were identified through the population-based Victorian Cancer Registry, capturing new diagnoses for two time periods: 2008–2009 and 2012–2013. Treatment was pre-emptively classified as ‘optimal’ or ‘suboptimal’, according to compliance with current treatment guidelines. Univariable and multivariable logistic regression models were fitted to determine factors associated with treatment and survival. *Results*: Altogether, 1442 DLBCL cases were included. Based on multivariable analysis, delivery of optimal treatment was less likely for those aged ≥80 years (*p* < 0.001), women (*p* = 0.012), those with medical comorbidity (*p* < 0.001), those treated in a non-metropolitan hospital (*p* = 0.02) and those who were ex-smokers (*p* = 0.02). Delivery of optimal treatment increased between 2008–2009 and the 2012–2013 (from 60% to 79%, *p* < 0.001). Delivery of optimal treatment was independently associated with a lower risk of death (hazard ratio (HR) = 0.60 (95% confidence interval (CI) 0.45–0.81), *p* = 0.001). *Conclusion*: Delivery of optimal treatment for DLBCL is associated with hospital location and category, highlighting possible demographic variation in treatment patterns. Together with an increase in the proportion of patients receiving optimal treatment in the more recent time period, this suggests that treatment decisions in DLBCL may be subject to non-clinical influences, which may have implications when evaluating equity of treatment access. The positive association with survival emphasizes the importance of delivering optimal treatment in DLBCL.

## 1. Introduction

Diffuse large B-cell lymphoma (DLBCL) is an aggressive form of non-Hodgkin lymphoma in which long term survival and ultimately cure is possible following optimal treatment with a rituximab-based, anthracycline-containing regimen such as rituximab, cyclophosphamide, doxorubicin, vincristine, prednisone (R-CHOP) [1]. Survival outcomes for patients diagnosed with DLBCL have improved over time despite the lack of introduction of any new therapeutic modalities since rituximab in 2001 [2], suggesting improvements in supportive care, earlier diagnosis or a progressive increase in adoption of R-CHOP over time could be additional contributing factors. In practice, a number of different immuno-chemotherapy regimens are used to treat patients with DLBCL, with variation from the recommended treatment explained by the need to avoid specific toxicities in patients with medical comorbidities or frailty. However other non-clinical factors that may influence treatment include socioeconomic status (SES), residential location, hospital size and ethnicity [3,4,5,6,7].

We sought to identify factors associated with the type of treatment planned and subsequently delivered for a cohort of patients with DLBCL identified from the population-based cancer Victorian Cancer Registry (VCR). While Victoria is Australia’s second most populace and most densely populated state, with the majority living in a metropolitan area, it also includes areas classified by the Australian Bureau of Statistics (ABS) as remote, outer regional and inner regional [8]. Australians living in regional and remote areas are less likely to visit their primary care practitioner or undergo some recommended cancer screening activities and have reduced access to specialized services such as cancer centers compared with their counterparts living in metropolitan centers [9,10]. Access to healthcare in Australia is provided via the national, federal government-funded Medicare system, while public hospitals provide inpatient and some ambulatory care in a model funded by each of the state governments. Most diagnostic services and treatments can be provided to patients without charge. In addition to these public hospitals, there are also privately-operated hospitals, in which care is generally reimbursed through patients’ optional personally-funded membership of a private health insurer. We sought to evaluate the influence of SES on the delivery of optimal treatment in the context of a population with universal access to healthcare. We hypothesized that patient area of residence (whether non-metropolitan or metropolitan) and type of hospital may influence treatment decisions and subsequently survival. 

## 2. Methods

### 2.1. Patient Cohort

All incident cases of pathologically confirmed DLBCL, occurring in patients aged 18 years and over, diagnosed and treated in Victoria, Australia (population 6.15 million) were identified through the population-based VCR. Data were collected covering two time periods: between 1st January 2008 and 31st December 2009 and between 1st January 2012 and 31st December 2013. Reporting of all new cancer cases by pathology laboratories and hospital medical record departments to the VCR is mandatory under state legislation. ICD-O3 codes 9680/9683 were used to identify cases. Subsequently, the diagnostic pathology report in the VCR patient file was reviewed to ensure they were correctly classified. The study was conducted with ethical approval from Cancer Council Victoria’s Human Research Ethics Committee (HREC) (HREC0911, date of approval 7th July 2010) and approval from hospital-specific HRECs.

### 2.2. Medical Record Review and Data Collection

Trained data managers attended the treating hospital of each patient and extracted predetermined clinical data through retrospective review of medical records. Electronic and paper medical records, specialist letters, chemotherapy charts, radiotherapy charts, pharmacy databases and pathology databases were reviewed wherever available. Medical comorbidities were assessed by review of medical records and assigned a score using the Adult Comorbidity Evaluation-27 (ACE-27) index, a validated tool for cancer patients that captures the presence and severity of comorbidity across multiple organ systems, enabling comparison between individuals (Table S1) [11,12]. Treatment center type was classified as public (government-funded hospital providing hospital treatment at no cost) or private hospital. Treatment location and patient area of residence were designated as metropolitan or non-metropolitan using the Area of Remoteness Index of Australia [8]. Referral source and type of specialist referred to (hematologist or medical oncologist) was recorded. Each patient’s residential location was used to determine an SES indicator score formulated by the ABS known as Socio-Economic Indexes for Areas from 0 (least disadvantaged) to 100 (most disadvantaged). Mortality data, including cause of death, were obtained via linkage to Victorian death records, the National Death Index or the ABS, with death data complete to 31st December 2016. 

### 2.3. Treatment Categories

Consistent with US and European guidelines, optimal treatment was defined as any one of the following regimens: R-CHOP 6–8 cycles with or without radiotherapy [1,13], R-CHOP-like 68 cycles (including R-miniCHOP consisting of reduced doses of cyclophosphamide doxorubicin and prednisone as well as capping of vincristine [14], rituximab, cyclophosphamide, etoposide, vincristine, prednisone [R-CEOP] [15] in which etoposide replaces doxorubicin and rituximab, cyclophosphamide, doxorubicin, etoposide, prednisone [R-CHEP] in which etoposide replaces vincristine), rituximab, hyperfractrionated cyclophosphamide, vincristine, doxorubicin, dexamethasone (R-hyperCVAD) 6 cycles [16], rituximab-dose adjusted etoposide, prednisone, vincristine, cyclophosphamide, doxorubicin (R-DA-EPOCH) 6 cycles [17], rituximab, methotrexate, doxorubicin, cyclophosphamide, vincristine, prednisone, bleomycin (R-MACOPB) [18] and rituximab, cyclophosphamide, doxorubicin, methotrexate alternating with ifosfamide, etoposide, cytarabine (R-CODOX-M IVAC) [19]. If disease stage was I–II, optimal treatment could also include R-CHOP ≥3 cycles with radiotherapy [1,13]. Suboptimal treatment was defined as one of following regimens: R-CHOP or R-CHOP variants <6 cycles (other than stage I–II as described above), or any other chemotherapy-immunotherapy regimen. Palliative treatment included the use of steroids alone, vincristine with or without steroids or no treatment. Chemotherapy dose reductions and/or modifications were not extracted from the chemotherapy administration charts.

Planned and delivered treatments were ascertained from medical records with planned treatment defined as the treatment plan documented before commencement of therapy and treatment delivered defined as the therapy that was administered. Patients who died before completing planned optimal treatment (within 6 months of diagnosis) were included in the optimal treatment delivered group to reduce survival bias within this group. 

### 2.4. Analyses

All patients with DLBCL were included in the descriptive statistics. Patients with palliative or no treatment were excluded from regression analyses. Logistic regression models were fitted to determine the variables associated with planned and delivered treatment and survival. Variables included factors that may be associated with DLBCL prognosis: age, sex, stage, presence of systemic symptoms (defined as presence of fever, weight loss, night sweats, fatigue, pruritus), lactate dehydrogenase (LDH) level, extranodal involvement of one or more sites, serum albumin, medical comorbidity as well as demographic and geographic factors like residential location (metropolitan or non-metropolitan), hospital type (private or public), hospital location (metropolitan or non-metropolitan), referral source (general practitioner, specialist physician, surgeon), type of specialist referred to (hematologist, medical oncologist), smoking status (never smoked, ex-smoker or current smoker), country of birth (English-speaking country or non-English speaking country) and SES score (quintiles). Univariable logistic regression models were fitted for each of the outcomes (planned treatment, delivered treatment) and the demographic and clinical variables. Cox proportional hazards models estimated hazard ratios for death (HR_death_) associated with treatment type. Survival from time of DLBCL diagnosis was estimated for each treatment group using Kaplan–Meier analysis. From univariable analyses, those with *p* < 0.1 were included in the multivariable models. All statistical tests were two sided, with *p* < 0.05 considered statistically significant. Statistical analyses were performed using Stata/MP version 14.2 (StataCorp LLC, College Station, TX, USA).

## 3. Results

A total of 1442 DLBCL cases were included in the study, 624 in study period one (2008–2009) and 818 in study period two (2012–2013). The demographic and clinical features of patients in the two study periods are presented in Table 1. Treatment center characteristics are summarized in Table 2. Of the 1442 patients, 310 (21%) had at least one site of extranodal involvement including seven with concurrent CNS and systemic involvement and 13 with primary CNS involvement. 

After excluding cases with palliative or no treatment, planned treatment was evaluated as optimal for 82% in 2008–2009 and 90% in 2012–2013 (*p* < 0.001). There was also a significant increase in the percentage of patients completing optimal treatment from 60% in 2008–2009 to 79% in 2012–2013 (*p* < 0.001) (Table S2). These increases were due mainly to an increase of optimal treatment in the oldest age group (≥80 years) for whom planned optimal treatment increased from 37% to 50% (*p* < 0.001) and delivered optimal treatment increased from 22% to 41% (*p* < 0.001) (data not shown).

Factors associated with a reduced likelihood of both planned and delivered optimal treatment in the univariable analysis were older age, the presence of any medical co-morbidity, treatment in a private hospital, treatment in a non-metropolitan hospital, being a former smoker and treatment during the earlier study period (Table S2). In the multivariable analyses, patients in the oldest age group of ≥80 years and the middle age group of 60–79 years as well as those with CNS involvement were more likely to have suboptimal treatment planned (Table 3). The presence of systemic symptoms at diagnosis and diagnosis in the later study period (2012–2013) were independently associated with increased likelihood of having optimal treatment planned. Delivery of optimal treatment was more likely for younger patients, men, those without medical comorbidities, those treated at a metropolitan hospital, ex- or current smokers and those diagnosed in the later study period (Table 3). 

Using Kaplan–Meier survival estimates, three-year and four-year overall survival (OS) (excluding palliative and no treatment) was 78% and 76%, respectively. In univariable Cox regression analysis, patients with planned optimal treatment had a lower risk of death than those who had planned suboptimal treatment (HR_death_ = 0.37 (95% CI 0.29–0.48), *p* < 0.001). Delivery of optimal versus suboptimal treatment was also associated with lower all-cause mortality (HR_death_ = 0.45, (95%CI 0.36–0.57), *p* < 0.001)). We also performed a conditional survival analysis including only those who survived at least six months after diagnosis to test for an effect of early deaths on the association with treatment type. When early deaths were accounted for in this manner, the association between delivery of optimal treatment and lower all-cause mortality remained (HR_death_ = 0.41, (95% CI 0.31–0.53), *p* < 0.001).

Other factors associated with increased risk of death in univariable analysis were older age (60–79 years and ≥80 years), elevated serum LDH, low serum albumin, the presence of any level of medical comorbidity and advanced stage disease. Separate multivariable analyses were performed with planned and delivered optimal treatment as a covariate to assess which factors were independently associated with survival for the planned and delivered optimal treatment groups. In the multivariable Cox regression analysis, both planned (Table 4) and delivered optimal treatment were independently associated with lower all-cause mortality. Other factors independently associated with all-cause mortality were older age, elevated serum LDH, low albumin, medical comorbidity and stage III/IV disease (Table 4). 

## 4. Discussion

This study reports the treatment patterns and survival for patients diagnosed with DLBCL in Victoria, Australia, evaluating the possible determinants of variations in patterns of care over time and within different demographic groups. The main findings were that, in addition to the expected impact of age and medical comorbidity, the factors associated with a reduced likelihood of completing optimal therapy were male sex, being diagnosed and treated in the earlier time period or being treated in a rural hospital. Delivery of optimal treatment was independently associated with increased survival, reinforcing the importance of identifying its determinants. 

Patients diagnosed with DLBCL in the earlier period were less likely to have planned optimal treatment than those diagnosed in 2012–2013; this was primarily driven by a change in treatment pattern for patients aged 80 years and over. This could reflect improvements in supportive care and changing patient attitudes towards receiving intensive therapy at an older age. It also likely reflects an increasing adoption of dose-modified aggressive treatment regimens for the elderly and it is noted that between the two study time periods a practice-changing paper outlining the use of dose-reduced R-CHOP known as R-miniCHOP for patients over 80 years became a standard of care in this group [14]. In our study, R-miniCHOP was classified as an optimal treatment however we were commenting specifically on the numbers of patients receiving R-miniCHOP or full-dose R-CHOP. 

Planned delivery of suboptimal treatment was more likely not only for the oldest age group ≥80 years but also for both the intermediate age group 60–80 years, independent of medical comorbidity. This raises a concern that age may be playing a larger role in treatment decisions than may be desirable, particularly in light of other population-based studies suggesting that optimal treatment, when applied judiciously, are appropriate for patients aged ≥80 years [20]. 

CNS involvement was independently associated with suboptimal treatment and this may be because patients with CNS involvement at diagnosis often have a lower performance status at diagnosis for which optimal treatment regimens may be contraindicated. In addition, there were a small number of patients included who had primary CNS involvement for which there is a lack of a satisfactory standard of care [21,22]. The presence of systemic symptoms at diagnosis was independently associated with a greater likelihood of optimal planned treatment, which is not a typical factor in treatment decision-making. In this study, the prevalence of systemic symptoms was high (67%) due to the definition of systemic symptoms that included the conventional constitutional symptoms of weight loss, sweats and fever as well as the non-conventional symptoms of fatigue and pruritus. The reason for the association of systemic symptoms as defined is still not clearly explained by our data, however a possible explanation is that the recording of systemic symptoms in clinical notes could be a surrogate for more rigorous documentation and that this feature may be associated with higher quality care. 

Delivery of optimal treatment was assessed separately, as successful treatment delivery is a reflection of whether the planned treatment is tolerated by the patient. We observed that smoking status was independently associated with a lower likelihood of delivery of optimal treatment, but not planning of optimal treatment. This could be because associated smoking-related respiratory or cardiac diseases that were not documented in the clinical notes became apparent during treatment and led to conversion of optimal planned treatment to suboptimal treatment delivered. Alternately, there could be other social or psychological factors within ex- and current smokers that make it less likely for them to complete treatment. Although this association was significant in the group of ex-smokers rather than current smokers, this is most likely due to the small numbers of current smokers in our cohort, rather than because of an effect limited to ex-smokers.

Delivery of optimal treatment was also less likely in non-metropolitan compared with metropolitan hospitals raising the possibility of treatment variation by hospital location. However, we did not demonstrate a difference in treatment by residential location, suggesting that some patients residing in non-metropolitan locations may seek treatment in metropolitan centers. This observation is in keeping with other studies suggesting patients treated in community hospitals in less urbanized areas hospitals received lower intensity chemotherapy and less radiotherapy than those treated in university hospitals [4,23]. In Australia, patients living in rural areas are reported to be less likely to receive chemotherapy for advanced stage colorectal cancer [24] however no similar studies in lymphoma have been published to date. Some recognized barriers for rural and remote practitioners to deliver cancer care according to guidelines in Australia include low caseloads resulting in less specialized experience and difficulty accessing specialized experts through mechanisms such as cancer multidisciplinary teams (MDTs) that are a standard feature of cancer care in larger hospitals [25]. Specialist outreach clinics, in which specialists visit non-metropolitan areas to provide input and support to local cancer services, are an evidence-based mechanism that improves health outcomes in non-metropolitan areas [26] and are in place in some regions of Victoria. A recent French study identified patients treated in high caseload hospitals and those whose cases were discussed in multidisciplinary meetings or were included in a clinical trial were associated with favourable survival [27]. Other factors in non-metropolitan hospitals that may limit delivery of optimal treatment include lack of appropriate supportive care infrastructure to underpin the safe delivery of aggressive chemotherapy, and patient or clinician preference. Other factors that may account for geographic disadvantage in cancer care delivery include SES, which was not a factor in our study or previous studies, and the presence of a larger indigenous population in some non-metropolitan areas, which we were not able to assess. 

Male sex was independently associated with a higher likelihood of delivery of optimal treatment, however other population-based studies report inconsistent results, with a large study of all types of B cell non-Hodgkin lymphoma showing no discrepancy in treatment by sex [28], while a smaller study of treatment of mantle cell lymphoma suggesting women were more likely to have received suboptimal therapy [29]. The observed treatment disparity in women is not explained by the variables we were able to measure in this study but may be related to other known gender disparities in patients’ experience of cancer treatment, such as the higher rate of anxiety and depression reported in women [30]. While we did not observe an association between sex and planned treatment nor for overall survival, the observation of any treatment variation by sex is concerning and warrants further investigation.

A reduced likelihood of optimal treatment delivered in private compared with public hospitals in the univariable analysis however this association was not maintained in the multivariable analysis (*p* = 0.09). It could be worth examining in a larger population, as in the Australian context routine use of cancer MDTs for hematological malignancies may not be as widespread as in the public (government) hospitals due to lower caseloads and smaller groups of specialists in practice in each private hospital location. 

The estimated overall survival in our study is in keeping with a Dutch study reporting a five-year OS of 63% for patients treated with R-CHOP [31]. We identified elevated serum LDH and advanced stage disease as being associated with reduced survival, in keeping with these factors remaining important components of the DLBCL International Prognostic Index (IPI) [32]. Low albumin, which is not part of the IPI, was also independently associated with reduced survival, consistent with the findings of other groups who have identified this as an additional independent prognostic marker [32,33,34].

Both planned optimal treatment and delivery of optimal treatment were associated with improved survival. This suggests two separate predictive factors for survival in DLBCL. First, the clinician assessment of a patient who is fit enough to have a plan of optimal treatment is an independent predictor of survival and second, the ability of the patient to complete the planned treatment is an additional predictor. Importantly, while advanced age (≥80 years) was independently associated with reduced survival, survival of the intermediate age group (60–79 years) was not different to the younger patients. 

Compared with other large population studies, we were able to report more detailed clinical data due to extensive review of the patients’ medical record [32,35,36]. The limitations of this study, related to the retrospective nature of the chart review, were that we were unable to report performance status nor other objective measures of frailty, or calculate the IPI and therefore our observations of reduced use of optimal treatment in elderly patients should be interpreted in the knowledge that comorbidity but not frailty were accounted for as potential confounders. We were also unable to report on dose variations of either chemotherapy or rituximab during treatment, which may have had an impact on survival, nor were we able to evaluate whether R-CHOP variants such as R-miniCHOP, which are considered optimal treatment in patients ≥80 years, were used according to the available evidence. The use of medical records as the source material means that data collected could be subject to bias if there were systematic differences in the way medical information was documented at different hospitals. 

## 5. Conclusions

The major finding was of increasing use of optimal treatment for DLBCL over time, particularly within the oldest age group. This encouraging finding suggests that clinicians are applying relevant new evidence for appropriate treatment of elderly patients in community practice. Overall, survival data in our study support the use of optimal treatment for all patient age groups. The less frequent use of optimal treatment in non-metropolitan hospitals remains a concern and although we did not observe an association with lower survival, this may be due to small numbers. Further evaluation is warranted to determine whether this difference is reproducible and whether it may be due to a disparity in health access, patient education or social factors. Our findings are likely to be representative of the delivery of optimal care to patients with other hematological malignancies. In light of the rapidly evolving standards of care in the treatment of hematological malignancies as a result of increasing availability of novel therapies, there is an urgent need to ensure adequate structures are in place to support rapid implementation of the latest evidence-based treatments. 

## Figures and Tables

**Table 1:** Demographics and clinical features of study participants overall and across the two study periods.

Patient Characteristics		Study Period				Total	
		2008/2009 (N = 624)		2012/2013 (N = 818)		(N = 1442)	
		N	(%)	N	(%)	N	(%)
Age	<60	184	(30)	195	(24)	379	(26)
	60–79	309	(50)	442	(54)	751	(52)
	≥80	131	(21)	181	(22)	312	(22)
Sex	Male	371	(60)	469	(57)	840	(58)
	Female	253	(41)	349	(43)	602	(42)
Stage	“Limited” I–II	221	(36)	296	(36)	517	(36)
	“Extensive” III–IV	299	(48)	440	(54)	739	(51)
	Not stated	104	(17)	82	(10)	186	(13)
Systemic symptoms *	No	31	(21)	295	(36)	425	(30)
	Yes	452	(72)	523	(64)	975	(68)
	Not stated	42	(6.7)	0	(0)	42	(2.9)
LDH	Normal	182	(29)	231	(28)	413	(29)
	Greater than ULN	323	(52)	321	(39)	644	(45)
	Not stated	119	(19)	266	(33)	385	(27)
Albumin *	Normal or high	417	(67)	466	(57)	883	(61)
	Less than LLN	179	(29)	315	(39)	494	(34)
	Not stated	28	(4.5)	37	(4.5)	65	(4.5)
Comorbidity	None	160	(26)	211	(26)	371	(26)
	Mild	418	(67)	562	(69)	980	(68)
	Moderate/Severe	46	(7.4)	45	(5.5)	91	(6.3)
Extranodal site involved	None or 1	509	(82)	639	(78)	1,148	(80)
	More than 1	115	(18)	179	(22)	294	(20)
Smoking status *	Never/Non	283	(45)	494	(61)	777	(54)
	Ex-smoker	136	(22)	209	(26)	345	(24)
	Current	70	(11)	66	(8.0)	136	(9.4)
	Not stated	135	(22)	49	(6.0)	184	(13)
Country of birth *	Australia	364	(58)	496	(61)	860	(60)
	Other	260	(42)	259	(32)	519	(36)
	Not stated	0	(0)	63	(7.7)	63	(4.4)
SES score *	Q1–most disadvantaged	158	(25)	147	(18)	305	(21)
	Q2	83	(13)	168	(21)	251	(17)
	Q3	96	(15)	153	(19)	249	(17)
	Q4	91	(16)	145	(18)	236	(16)
	Q5–least disadvantaged	193	(31)	135	(17)	328	(23)
	Not stated	3	(0.5)	70	(8.6)	73	(5.1)
Response to treatment	Complete/Near complete	426	(68)	478	(58)	904	(64)
	Partial response	38	(6)	40	(4.9)	78	(5.4)
	PD/SD	57	(9)	25	(3.1)	82	(5.7)
	Died ^†^	2	(0.3)	-	-	2	(0.1)
	Missing	101	(16)	275	(34)	376	(26)
Residential location	Metro	461	(74)	616	(75)	1,077	(75)
	Non-metropolitan	163	(26)	201	(25)	364	(25)
	Not stated	0	(0)	1	(0.1)	1	(0.01)

* *p* < 0.05 from chi^2^ test for association; ^†^ Not collected in the 12/13 dataset; LDH—lactate dehydrogenase, ULN—upper limit of laboratory normal range, LLN—lower limit of laboratory normal range, SES—socio-economic status, PD—progressive disease, SD—stable disease.

**Table 2:** Characteristics of treatment including referral pathways, type of treatment cancer and planned and delivered care overall and across study periods.

Treatment Characteristics		Study Period				Total	
		2008/2009 (N = 624)		2012/2013 (N = 818)		(N = 1442)	
		N	(%)	N	(%)	N	(%)
Treatment *	Any private	231	(37)	217	(27)	448	(31)
	Only public	393	(63)	515	(63)	908	(63)
	Not stated	0	0	86	(11)	86	(6.0)
Initial treatment location *	Any metropolitan location	540	(87)	671	(82)	1,211	(84)
	Non-metropolitan location	83	(13)	147	(18)	230	(16)
	Not stated	1	(0.2)	0	0	1	(0.1)
Referred by *	GP	257	(41)	371	(46)	628	(44)
	Physician/Emergency dept	124	(20)	81	(10)	205	(14)
	Surgeon	164	(26)	89	(11)	253	(18)
	Not stated	79	(13)	277	(34)	356	(25)
Referred to *	Hematologist	455	(73)	472	(58)	927	(64)
	Medical Oncologist	156	(25)	210	(26)	366	(25)
	Other	13	(2.1)	136	(17)	149	(10)
Planned treatment	Optimal	449	(72)	598	(73)	1,047	(73)
	Suboptimal	101	(16)	64	(7.8)	165	(11)
	Palliative/None	41	(6.6)	65	(8.0)	106	(7.4)
	Not stated	33	(5.3)	91	(11)	124	(8.6)
Delivered treatment	Optimal	323	(35)	507	(62)	830	(58)
	Suboptimal	217	(52)	136	(17)	353	(25)
	Palliative/None/Not stated	84	(13)	175	(21)	259	(18)

* p < 0.05 from chi^2^ test for association; GP—general practitioner.

**Table 3:** Factors associated with optimal treatment (multivariable). ^†^

Variable		Optimal Treatment Planned			Optimal Treatment Delivered		
		OR	95% CI	*p*	OR	95% CI	*p*
Age	<60	1			1		
	60–79	0.47	0.27–0.80	0.006	0.85	0.58–1.25	0.42
	≥80	0.09	0.0.5–0.15	<0.001	0.38	0.24–0.60	<0.001
Sex	Male			-	1		
	Female				0.68	0.28–0.92	0.012
Systemic symptoms	Yes	1					-
	No	2.03	1.38–2.98	<0.001			
No extranodal involvement		1					-
CNS involvement		0.27	0.11–0.70	0.007			
Comorbidity	None			-	1		
	Mild				0.49	0.33–0.73	<0.001
	Mod/Severe				0.53	0.28–1.02	0.057
Smoking status	Never			-			
	Current				0.98	0.60–1.61	0.95
	Previous				0.67	0.48–0.93	0.018
Treatment center type	Public			-	1		
	Private				0.76	0.56–1.04	0.09
Treatment location	Metropolitan			-			
	Non-metropolitan				0.63	0.44–0.92	0.015
Study period	2008–2009	1			1		
	2012–2013	2.61	1.80–3.78	<0.001	2.75	2.06–3.67	<0.001

^†^ combined data from study periods 1 and 2. OR—odds ratio, CI—confidence interval, CNS—central nervous system.

**Table 4:** Factors associated with all-cause mortality (multivariable). ^†^

	HR	95% CI	*p*
Optimal treatment delivered	0.60	0.45–0.81	0.001
Age 60–79	1.23	0.83–1.82	0.3
Age ≥80	1.23	1.92–4.47	<0.001
LDH high	1.74	1.26–2.40	0.001
Albumin low	2.00	1.49–2.66	<0.001
Comorbidity mild	1.97	1.27–3.04	0.002
Comorbidity Mod/severe	2.34	1.27–4.31	0.006
Stage III/IV	1.54	1.12–2.10	0.008

^†^ combined data from study periods 1 and 2. HR—hazard ratio, CI—confidence interval, LDH—lactate dehydrogenase.

## Supplementary Materials

The following are available online at https://www.mdpi.com/2072-6694/11/7/928/s1, Table S1: Comorbidities evaluated with the ACE-27 score, Table S2: Factors associated with optimal treatment (univariable).
