# Supplementary material for: The Use of Optimal Treatment for DLBCL Is Improving in All Age Groups and Is a Key Factor in Overall Survival, but Non-Clinical Factors Influence Treatment
Source: Cancers (Basel). 2019 Jul 2;11(7):928. doi: 10.3390/cancers11070928 (PMC6678990; doi:10.3390/cancers11070928)
Supplement: Supplementary file 1 [file cancers-11-00928-s001.pdf]

Supplementary Materials

Table S1. Comorbidities evaluated with the ACE-27 score.

Organ System †	Specific Comorbid Condition †
Cardiovascular System	Myocardial Infarct
	Angina/Coronary Artery Disease
	Congestive Heart Failure
	Arrhythmias
	Hypertension
	Venous Disease
	Peripheral Arterial Disease
Respiratory System	
Gastrointestinal System	
Hepatic	Stomach/Intestine
	Pancreas
Renal System	End-stage renal disease
Endocrine System	Diabetes Mellitus
Neurological System	Stroke
Dementia	
Paralysis	
Neuromuscular	
Psychiatric	
Rheumatologic	
Immunological System	AIDS
Malignancy	Solid Tumor
	Leukemia and Myeloma
	Lymphoma
Substance Abuse	Alcohol
	Illicit Drugs
Body Weight	Obesity
Overall Comorbidity Score ‡ = 0 (nil), 1 (mild), 2 (moderate), 3 (severe)	

† Each medical comorbidity is graded according to descriptive index where broadly Grade 1 = mild decompensation, Grade 2 = moderate decompensation, Grade 3 = severe decompensation. ‡ Overall comorbidity score is calculated by assigning a score equivalent to the highest-ranked single comorbidity. In cases where two or more comorbidities of moderate severity occur in different organ systems, the overall comorbidity score is assigned as severe. ACE-27—Adult Comorbidity Evaluation-27, AIDS—adult immunodeficiency syndrome. [11,12].

Table S2. Factors associated with optimal treatment (univariable).

		Planned Optimal Treatment			Planned Treatment Delivered		
		**OR †**	**(95% CI)**	***p*-value**	**OR †**	**(95% CI)**	***p*-value**
Age	<60 (Ref)	1			1		
	60–79	0.52	0.31–0.89	0.02	0.71	0.52–0.97	0.04
	≥80	0.10	0.06–0.18	<0.001	0.25	0.17–0.36	<0.001
Sex	Male (Ref)	1			1		
	Female	0.76	0.55–1.06	0.11	0.80	0.62–1.03	0.08
Stage	"Limited" I–II (Ref)	1			1		
	"Extensive" III–IV	1.21	0.84–1.74	0.31	1.02	0.78–1.33	0.91
Systemic symptoms	No (Ref)	1			1		
	Yes	1.49	1.06–2.12	0.02	1.04	0.79–1.37	0.78
LDH	Normal (Ref)	1			1		
	Greater than ULN	1.12	0.76–1.65	0.58	0.94	0.70–1.25	0.66
Albumin	Normal (Ref)	1			1		
	Lower than LLN (<37)	0.78	0.55–1.11	0.17	0.94	0.72–1.23	0.67
Comorbidity	None (Ref)	1			1		
	Mild	0.43	0.27–0.68	<0.001	0.42	0.31–0.59	<0.001
	Moderate/Severe	0.49	0.23–1.05	0.07	0.46	0.26–0.80	0.01
Residential location	Metro (Ref)	1			1		
	Non-metropolitan	1.06	0.72–1.55	0.77	0.88	0.66–1.17	0.37
Treatment	Only public (Ref)	1			1		
	Any private	0.61	0.43–0.85	0.004	0.68	0.53–0.89	0.004
Initial treatment location	Any metro (Ref)	1			1		
	Non-metropolitan location	0.63	0.42–0.94	0.03	0.68	0.49–0.95	0.02
Referred by	GP (Ref)	1			1		
	Physician/Emergency dept	0.78	0.48–1.26	0.30	0.90	0.62–1.31	0.59
	Surgeon	0.82	0.52–1.27	0.37	0.79	0.57–1.11	0.18
Referred to	Hematologist (Ref)	1			1		
	Medical Oncologist	0.79	0.55–1.13	0.19	1	0.75–1.33	0.99
Extranodal site involved‡	None or 1 (Ref)	1			1		
	More than 1	0.90	0.61–1.34	0.62	0.87	0.65–1.18	0.37
Smoking status	Never/Non (Ref)	1			1		
	Ex-smoker	0.64	0.43–0.96	0.03	0.63	0.47–0.85	0.003
	Current	0.94	0.51–1.73	0.85	1.13	0.72–1.77	0.60
Country of birth	Australia (Ref)	1			1		
	Other	0.91	0.65–1.28	0.59	0.86	0.66–1.12	0.26
SES score	Q1—Most disadvantaged	1.25	0.78–1.98	0.35	1.09	0.76–1.57	0.65
	Q2	1.40	0.85–2.31	0.19	1.42	0.96–2.10	0.08
	Q3	1.41	0.85–2.32	0.18	1.41	0.95–2.08	0.09
	Q4	2.63	1.43–4.82	0.002	1.55	1.04–2.33	0.03
	Q5—Least disadvantaged 9 (Ref)	1			1	0.64–1.32	0.65
Study period	2008–2009	1			1		
	2012–2013	2.10	1.50–2.94	<0.001	2.50	1.94–3.23	<0.001

† From univariable logistic regression model: binary outcome; odds of optimal treatment vs suboptimal treatment; ‡ association between treatment type and individual extranodal sites tested and any site with $p < 0.1$ included in multivariable analysis; Ref—Reference variable, LDH—lactate dehydrogenase, ULN—upper limit of laboratory normal range, LLN—lower limit of laboratory normal rangeSES—socio-economic status.
